# Neither ultrasound synovitis nor clinical-ultrasound phenotypes of established rheumatoid arthritis predict response to targeted therapy

**DOI:** 10.1093/rheumatology/keaf315

**Published:** 2025-06-25

**Authors:** John Fitton, Andrew Melville, Andrea Di Matteo, Jackie Nam, Shouvik Dass, Benazir Saleem, Rudresh Shukla, Paul Emery, Richard J Wakefield, Elizabeth M A Hensor, Maya H Buch

**Affiliations:** Leeds Biomedical Research Centre, National Institute for Health and Care Research, Leeds, UK; Department of Rheumatology, Chapel Allerton Hospital, Leeds Teaching Hospitals NHS Trust, Leeds, UK; Leeds Biomedical Research Centre, National Institute for Health and Care Research, Leeds, UK; Leeds Biomedical Research Centre, National Institute for Health and Care Research, Leeds, UK; Leeds Biomedical Research Centre, National Institute for Health and Care Research, Leeds, UK; Department of Rheumatology, Chapel Allerton Hospital, Leeds Teaching Hospitals NHS Trust, Leeds, UK; Department of Rheumatology, Chapel Allerton Hospital, Leeds Teaching Hospitals NHS Trust, Leeds, UK; Department of Rheumatology, Chapel Allerton Hospital, Leeds Teaching Hospitals NHS Trust, Leeds, UK; Centre for Musculoskeletal Research, School of Biological Sciences, Faculty of Biology, Medicine and Health, University of Manchester, Manchester, UK; NIHR Manchester Biomedical Research Centre, Manchester University NHS Foundation Trust, Manchester Academic Health Science Centre, University of Manchester, Manchester, UK; Leeds Biomedical Research Centre, National Institute for Health and Care Research, Leeds, UK; Leeds Institute of Rheumatic and Musculoskeletal Medicine, University of Leeds, Leeds, UK; Leeds Biomedical Research Centre, National Institute for Health and Care Research, Leeds, UK; Leeds Institute of Rheumatic and Musculoskeletal Medicine, University of Leeds, Leeds, UK; Leeds Biomedical Research Centre, National Institute for Health and Care Research, Leeds, UK; Leeds Institute of Rheumatic and Musculoskeletal Medicine, University of Leeds, Leeds, UK; Centre for Musculoskeletal Research, School of Biological Sciences, Faculty of Biology, Medicine and Health, University of Manchester, Manchester, UK; NIHR Manchester Biomedical Research Centre, Manchester University NHS Foundation Trust, Manchester Academic Health Science Centre, University of Manchester, Manchester, UK; Leeds Institute of Rheumatic and Musculoskeletal Medicine, University of Leeds, Leeds, UK

**Keywords:** rheumatoid arthritis, phenotypes, ultrasound, power Doppler, synovitis, DMARD, response

## Abstract

**Objectives:**

To identify patient sub-phenotypes using clinical and imaging measures in established rheumatoid arthritis (RA) and to establish if baseline ultrasound synovitis and/or baseline patient sub-phenotypes predicts response to targeted therapy (TT).

**Methods:**

This was an observational cohort study of consecutively recruited patients with established RA starting TT. Participants received clinical assessment, 38-joint musculoskeletal ultrasound (MSUS), measuring grey scale and power Doppler (PD) synovitis/tenosynovitis, and patient reported outcomes (PRO), prior to and 6 months after treatment. Latent profile analysis of the clinical and MSUS variables identified disease clusters, and multinomial logistic regression models determined whether these and/or baseline synovitis predicted EULAR response.

**Results:**

Of 200 recruited patients, three clusters, low to high inflammatory, were identified with median (IQR) total joint PD 2 (0–4) in cluster 1, 9 (6–13) in cluster 2 and 29.5 (21–45) in cluster 3. A health assessment questionnaire correlated with disease activity, and DAS-P score distributions differed between clusters (*P* = 0.001) but with identical medians. Other PROs did not differ. At 6 months relative risk ratio of EULAR response in those with baseline synovitis compared with those without was 1.05 (95% CI: 0.38, 2.93) and response compared with the lowest inflammatory cluster [1] was 1.50 (95% CI: 0.61, 3.70) for cluster 2 and 1.24 (95% CI: 0.43, 3.60) for cluster 3.

**Conclusion:**

This is the first study to identify RA phenotypes employing 38-joint MSUS. Strikingly, neither baseline synovitis nor inflammatory cluster predicted clinical response. Mechanistic understanding of TT response/non-response is needed and clinical trials need to still capture all these populations.

Rheumatology key messages

- Patients in a cluster with high DAS28 and low MSUS inflammation respond to targeted therapies.
- Baseline ultrasound power Doppler synovitis does not predict response to targeted therapy in RA.
- Excluding RA patients in low inflammatory clusters from trials is premature as many respond.

## Introduction

The treatment of rheumatoid arthritis (RA) has been revolutionized by the availability of targeted therapies (TT) comprising biologic DMARDs (bDMARDs) and Janus kinase inhibitor (JAKi) targeted synthetic (ts) DMARDS. RA is a heterogeneous condition with a complex pathogenesis, and the mechanisms underlying treatment response and failure are likely no less complex [1, 2].

Clinical studies have identified different RA phenotypes, highlighting the heterogeneity of the RA population [3, 4]. These have focused on clinical and patient reported outcome (PRO) data, showing high inflammatory to minimal or no inflammatory clusters of disease activity, with respective patterns of PROs including low disease activity, but high PRO outcomes of pain, fatigue and psychological distress [4]. None of the work published to date has used musculoskeletal ultrasound (MSUS) to validate these subgroups for differences in the presence of synovitis or non-synovial inflammation and/or wider joint pathology. The absence of MSUS power Doppler (PD) is used to equate with the absence of inflammatory disease amenable to treatment, but studies confirming this notion are lacking.

This study aimed to identify phenotypes of patients with established RA starting a first or subsequent TT, using clinical characteristics and extensive (38 joint) MSUS measures that were evaluated against PROs and to determine whether the presence of baseline US-detected synovitis and/or baseline disease cluster predicted response to therapy.

## Methods

### Study design

This prospective, longitudinal, observational study was conducted at Leeds Teaching Hospitals NHS Trust (LTHT), a tertiary centre with dedicated TT clinics comprising over 2000 RA patients. Ethical approval was granted under the RADAR (Rheumatoid Arthritis DiseAse Research) protocol (09/H1307/98). Written consent to the RADAR study was obtained for all patients included within the manuscript. This study was part of a research programme that was subject to patient and public involvement and input.

### Target population: inclusion/exclusion criteria

All patients with RA starting one of the approved TTs in line with standard of care between February 2018 and March 2020 were screened for recruitment. Key inclusion criteria included (i) age ≥18 years, (ii) a confirmed diagnosis of RA according to ACR/EULAR 2010 criteria, and (iii) one of high disease activity (disease activity score for 28 joints [DAS28] > 5.1) despite treatment with ≥2 conventional synthetic DMARDs as per National Institute for Health and Care Excellence guidance [5], primary non-response to a TT (failure to achieve an improvement in DAS28 ≥ 1.2 at 6 months, or 3 months if clear non-response in line with usual practice) or secondary failure of TT determined by the treating rheumatologist. Patients were excluded if taking ≥10 mg oral prednisolone and/or equivalent alternative glucocorticoid and/or exposure to intramuscular corticosteroid within the last 6 weeks.

### Clinical data collection and assessments

Demographic information, past medical history and details of past TT were recorded. Patients had clinical assessments at baseline, week 12 and week 24. Tender and swollen joint counts, patient general health visual analogue score (VAS) and inflammatory markers (CRP and ESR) were recorded at each visit and used to calculate DAS28-CRP and DAS28-ESR, as well as DAS-P, a derived proportion of the patient reported components of the DAS28 score (tender joint count [TJC], VAS) [6] and DAS28-CRP-2C (calculated using CRP and swollen joint count [SJC] and shown to reflect MSUS-identified joint synovitis better [7]). Response status was calculated at weeks 12 and 24 using DAS28-CRP scores and EULAR definitions of good and moderate response [8].

PRO data collected at baseline and weeks 12 and 24 comprised: Health Assessment Questionnaire—Disability Index (HAQ-DI), Functional Assessment of Chronic Illness Therapy (FACIT)-fatigue, and Hospital Anxiety and Depression Scale (HADS).

### Imaging assessments

X-rays of hands and feet at baseline were taken if previous X-rays did not show erosive changes and/or if X-ray of the hands and feet had not been undertaken within the previous 12 months. X-rays were checked for the presence or absence of erosions.

MSUS was performed by experienced sonographers blinded to clinical data, at baseline and 6 months as standard and at 3 months if the individual was deemed to be failing treatment necessitating switch in therapy. Thirty-eight joints (bilateral wrists, MCPs 1–5, PIPs 1–5, elbows, knees, tibiotalar joints, MTPs 1–5) were scanned from a dorsal view scored in a longitudinal plane with the transverse plane to confirm pathology and obtain grey scale (GS) synovitis and power Doppler (PD) scores (0–3) using the OMERACT–EULAR semi-quantitative scoring system [9, 10]. Clinically relevant synovitis was defined as PDUS ≥2 in at least one joint or total PD score >3 (if no single joint had PD ≥2) based on a study by Padovano *et al.* evaluating the prevalence of MSUS abnormalities in healthy subjects without inflammatory arthritis [11]. In addition, 20 tendons/tendon groups (bilateral wrist extensor compartments 2, 4 and 6, finger flexors 2, 3, 4, 5, tibialis posterior, peroneus longus and brevis) were scanned for GS and PD and based on the OMERACT–EULAR protocol [12]. Ten entheseal sites (bilateral lateral epicondyle of the elbows, patella ligament proximal insertion, patella ligament distal insertion, Achilles tendon, plantar fascia) were also scanned and scored using the OMERACT–EULAR scoring system [13]. Enthesis thickening was measured against a standardized set of measurements based on the Glasgow entheseal scoring system [14] for the lower limb and data on average ultrasound characteristics of the common extensor tendon insertion for the lateral epicondyle [15]. The presence of erosions, osteophytes and joint subluxation was recorded, but not formally scored (see Supplementary Table S1, available at *Rheumatology* online for measurements used).

The treating clinician was not informed of the MSUS findings and no treatment decisions were taken based on scan results. Ultrasound was performed on a General Electric (GE; GE HealthCare, Chicago, IL, USA) Logiq E9 machine with linear ML 6–15 MHz transducer and hockey stick linear array 8–18 MHz transducer.

### Statistical analysis

Latent profile analysis (LPA) was used to identify disease clusters, using baseline data: DAS28 components and MSUS scores (total joint, tendon and enthesis GS and PD scores; number of joints with erosions/osteophytes/subluxed joints/thickened tendons; number of thickened entheses/entheses with erosions/entheses with enthesophytes/entheses with calcifications). Age, sex, HADS anxiety and FACIT-F fatigue score were specified *a priori* as covariates in the LPA models. Poisson and negative binominal models were run for the distributions of the different clinical and ultrasound components to determine the best distributions for model convergence and fit. Models were run for two to five clusters and model fit was compared using the Bayesian information criterion (BIC) and Akaike’s information criterion (AIC).

The Kruskal–Wallis (K-W) test was used to compare differences in clinical and ultrasound variables between the latent classes identified, and Dunn’s test was used on variables with statistically significant K-W results. The chi square test was used for differences between categorical variables. All analyses were run in Stata/MP 16.0 (StataCorp LLC, College Station, TX, USA).

### Sample size

A minimum sample size of 200 patients was set based on simulation studies that showed that the BIC correctly identified the number of clusters present at *n* = 200 74% of the time when 10 items were included and the structure was complex (with no indicators that uniquely differentiated between clusters), and 99% of the time when 15 items were included and the structure was simple [16].

Due to the COVID-19 pandemic, follow-up data could only be obtained in 134/200 recruited patients at 3-months follow-up and 111 patients at 6 months, with 99 able to attend for MSUS before research was paused. To model DAS scores and the proportion of EULAR responders at 3 and 6 months, multiple imputation was used to address all missing baseline covariates and missing longitudinal DAS component values, except those resulting from cancelled visits during COVID-19 lockdowns, which were assumed to be missing completely at random and were therefore excluded. Multiple imputation via chained equations was used to create 50 complete datasets. For continuous interval, bounded and ordinal variables, predictive mean matching with 10 nearest neighbours was used in the imputation model; for binary variables, logistic regression was used. Details of imputed variables and models are included in the supplementary material, available at *Rheumatology* online. Following imputation, DAS scores and responder status were re-calculated and results of analyses in the imputed data were combined according to Rubin’s rules. Sensitivity analyses used single imputation of response or non-response for missing data instead of multiple imputation.

Responder status at 3 and 6 months was compared between the latent classes identified at baseline, using multinomial logistic regression models in which those who responded and those who experienced adverse events were compared with non-responders.

## Results

### Recruitment

A total of 200 patients were recruited. One hundred and ninety-six patients attended for full baseline investigations. The remaining four patients did not attend MSUS appointment but had all other baseline investigations and were included in the analysis.

### Baseline characteristics

Of 200 patients recruited, 150 (75%) were female, mean age was 56.8 years (s.d. 12.6; range 26.7– 88.7), mean disease duration 11.36 years (s.d. 8.01) and 152 (76%) patients were seropositive. Table 1 details the clinical and disease variables that informed the LPA, covariates and descriptive summary statistics of other characteristics, including prior TT exposure and reasons for TT failure. Fifty-eight (29%) of the cohort were TT-naïve and 88 (44%) met the EULAR definition of difficult to treat RA [17]. Supplementary Tables S2 and S3, available at *Rheumatology* online, show the number of patients exposed to each number of previous TT and each number of classes of therapy respectively. Supplementary Table S4, available at *Rheumatology* online, shows subsequent TT prescribed during the study. One hundred and fifty-four (78.6%) patients met the pre-determined criteria for clinically significant ultrasound-detected synovitis at baseline (PDUS ≥ 2 in at least one joint or total PD > 3 if no joint has PD ≥ 2).

**Table 1.** Baseline characteristics

	Combined clinical/ultrasound latent profile					
	**1** (***n* = 76**)	**2** (***n* = 82**)	**3** (***n* = 42**)	**Total** (***n* = 200**)	**χ^2^** ^a^	*P*
LPA indicators, median (IQR)						
TJC	12.0 (6.5, 16.5), *n* = 76	11.0 (8.0, 16.0), *n* = 82	15.0 (12.0, 21.0), *n* = 42	12.0 (8.0, 17.0), *n* = 200	8.96	**0.011**
SJC	3.0 (1.0, 5.0), *n* = 76	5.0 (3.0, 7.0), *n* = 82	9.0 (6.0, 12.0), *n* = 42	4.5 (2.0, 7.5), *n* = 200	69.79	**<0.001**
CRP	2.0 (2.0, 9.2), *n* = 76	6.7 (2.0, 20.0), *n* = 82	33.0 (16.3, 55.0), *n* = 42	8.3 (2.0, 25.0), *n* = 200	58.27	**<0.001**
VAS	70.0 (60.0, 80.0), *n* = 76	75.0 (65.0, 85.0), *n* = 82	79.5 (65.0, 80.0), *n* = 42	75.0 (62.5, 80.5), *n* = 200	4.88	0.087
Total joint GS	26.0 (20.0, 31.5), *n* = 72	36.5 (29.0, 43.0), *n* = 82	62.5 (51.0, 71.0), *n* = 42	34.5 (25.0, 45.0), *n* = 196	108.13	**<0.001**
Total joint PD	2.0 (0.0, 4.0), *n* = 72	9.0 (6.0, 13.0), *n* = 82	29.5 (21.0, 45.0), *n* = 42	6.5 (3.0, 18.0), *n* = 196	143.37	**<0.001**
Number of joints with erosions	1.0 (0.0, 2.0), *n* = 72	2.0 (1.0, 4.0), *n* = 82	5.0 (3.0, 8.0), *n* = 42	2.0 (1.0, 4.0), *n* = 196	60.32	**<0.001**
Number of joints with osteophytes	2.0 (1.0, 6.0), *n* = 72	3.0 (1.0, 7.0), *n* = 82	3.0 (1.0, 6.0), *n* = 42	3.0 (1.0, 6.0), *n* = 196	0.62	0.733
Number of subluxed joints	3.0 (0.0, 6.0), *n* = 72	3.5 (0.0, 7.0), *n* = 82	4.5 (1.0, 7.0), *n* = 42	3.0 (0.0, 6.0), *n* = 196	2.35	0.309
Total tendon GS	1.0 (0.0, 2.0), *n* = 72	5.0 (3.0, 7.0), *n* = 82	11.0 (7.0, 18.0), *n* = 42	4.0 (1.0, 8.0), *n* = 196	120.78	**<0.001**
Total tendon PD	0.0 (0.0, 0.0), *n* = 72	3.0 (0.0, 5.0), *n* = 82	6.0 (4.0, 13.0), *n* = 42	1.0 (0.0, 5.0), *n* = 196	106.64	**<0.001**
Number of thickened tendons	0.0 (0.0, 1.0), *n* = 71	0.0 (0.0, 1.0), *n* = 82	0.0 (0.0, 1.0), *n* = 42	0.0 (0.0, 1.0), *n* = 195	6.05	**0.049**
Total enthesis GS	2.0 (1.0, 4.0), *n* = 72	2.0 (1.0, 4.0), *n* = 82	4.0 (2.0, 6.0), *n* = 42	3.0 (1.0, 4.0), *n* = 196	10.44	**0.005**
Total enthesis PD	0.0 (0.0, 0.0), *n* = 72	0.0 (0.0, 0.0), *n* = 82	0.0 (0.0, 1.0), *n* = 42	0.0 (0.0, 0.5), *n* = 196	9.42	**0.009**
Number of thickened entheses	2.0 (1.5, 3.0), *n* = 72	2.0 (2.0, 3.0), *n* = 82	3.0 (2.0, 4.0), *n* = 42	2.0 (2.0, 3.0), *n* = 196	9.63	**0.008**
Number of entheses with erosions	0.0 (0.0, 0.0), *n* = 72	0.0 (0.0, 1.0), *n* = 82	1.0 (0.0, 1.0), *n* = 42	0.0 (0.0, 1.0), *n* = 196	18.25	**<0.001**
Number of entheses with enthesophytes	1.0 (0.0, 2.0), *n* = 72	2.0 (0.0, 2.0), *n* = 82	1.5 (0.0, 4.0), *n* = 42	1.0 (0.0, 2.0), *n* = 196	1.44	0.486
Number of entheses with calcifications	0.0 (0.0, 1.0), *n* = 72	0.0 (0.0, 1.0), *n* = 82	0.0 (0.0, 0.0), *n* = 42	0.0 (0.0, 1.0), *n* = 196	12.88	**0.002**
LPA covariates						
Age, years	54.7 (48.8, 61.4), *n* = 76	57.1 (47.9, 63.1), *n* = 82	62.2 (54.5, 72.2), *n* = 42	57.2 (49.8, 65.3), *n* = 200	8.97	**0.011**
Sex female, *n* (%)	64/76 (84.2%)	61/82 (74.4%)	25/42 (59.5%)	150/200 (75.0%)	8.82	**0.012**
HADS anxiety	9.0 (6.0, 14.0), *n* = 76	9.0 (5.0, 11.0), *n* = 82	7.0 (4.0, 13.0), *n* = 41	9.0 (5.0, 12.0), *n* = 199	1.46	0.482
FACIT-fatigue	17.5 (10.0, 25.5), *n* = 76	21.5 (12.0, 29.0), *n* = 82	15.5 (8.5, 25.5), *n* = 40	18.5 (10.0, 28.0), *n* = 198	4.04	0.133
Baseline variables not included in LPA						
Ever smoker, *n* (%)	25/63 (39.7%)	34/74 (45.9%)	24/37 (64.9%)	83/174 (47.7%)	6.08	**0.048**
Disease duration, years	10.0 (5.0, 15.5), *n* = 64	8.0 (4.0, 16.0), *n* = 69	13.0 (6.5, 20.0), *n* = 40	10.0 (5.0, 17.0), *n* = 173	6.80	**0.033**
Seropositivity, n (%)					23.89	**0.001**
RF and ACPA positive	24/76 (31.6%)	46/82 (56.1%)	32/42 (76.2%)	102/200 (51.0%)		
ACPA positive only	18/76 (23.7%)	12/82 (14.6%)	3/42 (7.1%)	33/200 (16.5%)		
RF positive only	9/76 (11.8%)	5/82 (6.1%)	3/42 (7.1%)	17/200 (8.5%)		
RF and ACPA negative	25/76 (32.9%)	19/82 (23.2%)	4/42 (9.5%)	48/200 (24.0%)		
ANA positive	6/76 (7.9%)	11/82 (13.4%)	4/42 (9.5%)	21/200 (10.5%)	1.33	0.514
X-ray erosions present, *n* (%)	26/60 (43.3%)	40/77 (51.9%)	22/39 (56.4%)	88/176 (50.0%)	1.82	0.402
ESR	10.0 (3.0, 21.0), *n* = 69	19.5 (8.0, 30.0), *n* = 72	35.0 (23.0, 66.0), *n* = 38	18.0 (7.0, 31.0), *n* = 179	39.84	**<0.001**
HADS depression	8.0 (5.0, 12.0), *n* = 76	8.0 (5.0, 11.0), *n* = 82	10.0 (5.0, 12.0), *n* = 41	8.0 (5.0, 12.0), *n* = 199	1.20	0.548
HAQ-DI	1.8 (1.2, 2.1), *n* = 76	1.6 (1.1, 2.1), *n* = 80	2.0 (1.8, 2.5), *n* = 41	1.8 (1.2, 2.1), *n* = 197	7.95	**0.019**
DAS28-CRP	4.9 (4.2, 5.5), *n* = 76	5.3 (4.8, 5.7), *n* = 82	6.2 (5.8, 6.7), *n* = 42	5.3 (4.7, 5.9), *n* = 200	50.85	**<0.001**
DAS28-CRP-P	0.6 (0.5, 0.6), *n* = 76	0.6 (0.5, 0.6), *n* = 82	0.5 (0.5, 0.6), *n* = 42	0.6 (0.5, 0.6), *n* = 200	31.01	**<0.001**
DAS28-CRP-2C	2.4 (2.1, 3.2), *n* = 76	3.5 (2.9, 4.1), *n* = 82	5.1 (4.4, 5.7), *n* = 42	3.3 (2.4, 4.3), *n* = 200	91.19	**<0.001**
No. of previous treatments	2.0 (1.0, 3.0), *n* = 76	1.0 (0.0, 2.0), *n* = 82	2.0 (0.0, 4.0), *n* = 42	1.0 (0.0, 3.0), *n* = 200	5.35	0.069
No. of previous classes	2.0 (1.0, 2.0), *n* = 75	1.0 (0.0, 2.0), *n* = 82	1.5 (0.0, 3.0), *n* = 42	1.0 (0.0, 2.0), *n* = 199	5.09	0.079
Descriptive-only summaries, *n* (%)						
DAS28-CRP > 3.2	75/76 (98.7%)	81/82 (98.8%)	42/42 (100.0%)	198/200 (99.0%)		
DAS28-ESR > 3.2	61/69 (88.4%)	71/72 (98.6%)	38/38 (100.0%)	170/179 (95.0%)		
Clinical synovitis (PD ≥ 2, total PD > 3)	34/72 (47.2%)	78/82 (95.1%)	42/42 (100.0%)	154/196 (78.6%)		
Reason for previous TT failure, *n* (%)						
First line	13 (17.1%)	30 (36.6%)	15 (35.7%)	58 (29%)		
Primary NR	17 (22.4%)	14 (17.1%)	11 (26.1%)	42 (21%)		
Secondary NR	39 (51.3%)	33 (40.2%)	11 (26.1%)	83 (41.5%)		
AE	7 (9.2%)	5 (6.1%)	5 (11.9%)	17 (8.5%)		

Demographics, serology, clinical findings and patient reported outcome measures are included for each cluster identified in the combined clinical and ultrasound three-class model.

^a^ Kruskal–Wallis test performed to identify differences between clusters for continuous variables, χ^2^ for categorical variables. Significant differences highlighted in bold. AE: adverse event; DAS28: disease activity score for 28 joints; FACIT: Functional Assessment of Chronic Illness Therapy; GS: grey scale; HADS: Hospital Anxiety and Depression Scale; HAQ-DI: Health Assessment Questionnaire—Disability Index; LPA: latent profile analysis; NR: non-response; PD: power Doppler; SJC: swollen joint count; TJC: tender joint count; TT: targeted therapy; VAS: visual analogue score.

### Clustering of patients using DAS28 and MSUS characteristics

LPA using DAS28 component scores and MSUS variables was performed and the BIC minimized at three classes, favouring this solution (fit statistics shown in Supplementary Fig. S1, available at *Rheumatology* online). For most participants in each class (82.9%, 86.6% and 85.0% of classes 1–3 respectively), the posterior probability of belonging to the assigned class was ≥90%, indicating stable class membership.

**Figure 1.**
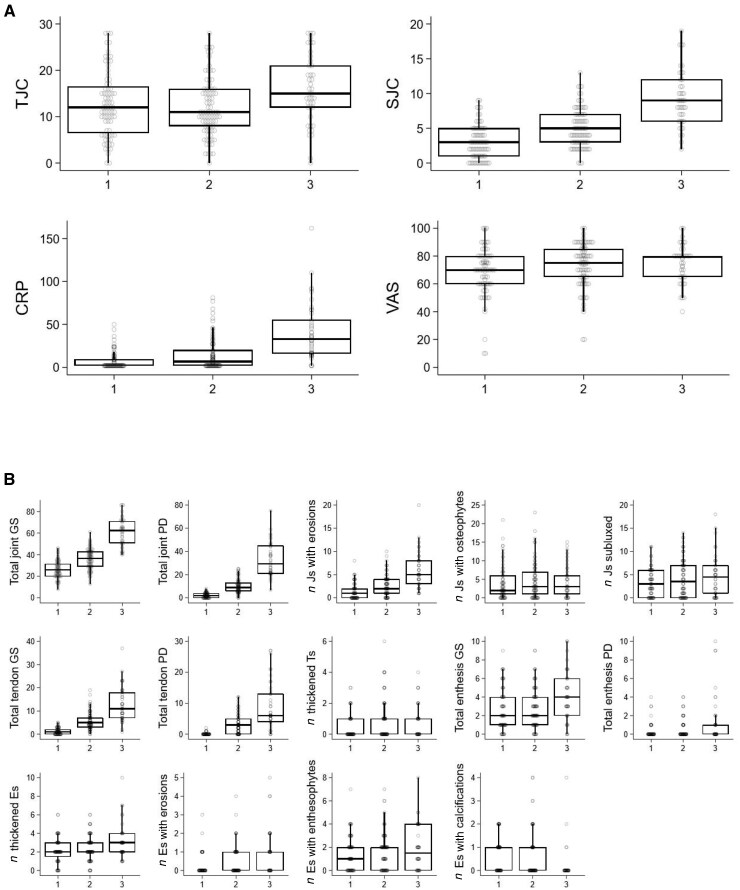
DAS28 component scores and ultrasound measures for each cluster. Box and whisker plots showing (**A**) DAS28 component scores and (**B**) individual ultrasound measures for each cluster (*x*-axis: 1–3). DAS28: disease activity score for 28 joints; E: enthesis; J: joint; GS: grey scale; PD: power Doppler; SJC: swollen joint count; T: tendon; TJC: tender joint count; VAS: visual analogue score

Cluster 1 comprised more female and seronegative individuals than the other clusters. Cluster 3 was the most inflammatory cluster as evidenced by higher DAS28-CRP, DAS28-CRP-2C, SJC, acute phase markers and PD synovitis, and comprised more seropositive and older age individuals. Cluster 3 had a significantly longer disease duration on Dunn testing (Table 1). Fig. 1A shows progression in SJC and CRP components of the DAS28 score across the clusters with subsequent higher baseline DAS28 scores. Dunn tests confirmed significant differences between all the clusters for SJC, CRP and ESR (Supplementary Table S5, available at *Rheumatology* online).

Table 1 and Fig. 1B show MSUS measures in each cluster, confirming increasing joint level inflammation from cluster 1 to cluster 3 defined by joint and tendon GS and PD. Significant differences were found for total joint GS and PD scores and number of joints with US erosions, although there was no statistical difference in the proportions of participants with X-ray erosions present at baseline. Tendon GS and PD were significantly different and, again, progressed from cluster 1 through to cluster 3. Significantly greater enthesis thickening, tendon thickening, enthesis Doppler, hypoechogenicity and erosions were observed in cluster 3 compared with other clusters, but more calcification in clusters 1 and 2 (Supplementary Table S6, available at *Rheumatology* online).

The only PRO (Table 1) to show a meaningful significant difference between the clusters was the HAQ-DI score, with cluster 3 having a significantly higher HAQ-DI than either cluster 1 or 2 (1/3 *P* = 0.008, 2/3 *P* = 0.004). There were no clinically relevant or statistically significant differences in anxiety or fatigue. DAS28-CRP-2C increased from cluster 1 to cluster 3 and the distributions of DAS-P differed, with higher values in the less inflammatory clusters (1/3 *P* < 0.001, 2/3 *P* = 0.001), although the medians did not differ.

### Baseline synovitis, latent profile and response to therapy

EULAR response status at 3 and 6 months was evaluated in those with and without clinically significant synovitis on MSUS and between the latent classes identified at baseline. Response to therapy in those with baseline erosive change was also assessed. Twelve patients within the cohort were determined to be failing treatment at 3 months warranting an immediate switch in therapy. In the imputed data, an estimated 69% of patients with clinically relevant synovitis responded to therapy at 3 months compared with 65% without clinically relevant synovitis (Table 2). No clinically or statistically significant difference in response between those with clinically significant US-detected synovitis on MSUS at baseline and those without was recorded. Neither was a difference identified between those with and without baseline erosion. A numerical increased relative risk for response in clusters 2 and 3 compared with cluster 1 was observed at 3 and 6 months but with wide confidence intervals meaning no statistically significant difference. No significant difference in the occurrence of AEs was observed. The models containing either clinically relevant US-detected synovitis or the latent classes did not differ from empty (null) models across any of the 50 imputed datasets, at either 3 or 6 months, and all Nagelkerke pseudo-*R*^2^ values were close to zero. Including additional predictors (age, sex, anti-CCP status, disease duration, number of previous treatments, smoker (ever), and whether the current treatment was a JAKi) did not alter these findings, nor did sensitivity analyses using single imputation (data not shown).

**Table 2.** Response outcomes

	Responded, %	RRR (95% CI)	AE, %	RRR (95% CI)
Response at 3 months (*n* = 164)				
MUS synovitis at BL				
No (estimated 21%)	65	Reference	6	Reference
Yes (estimated 79%)	69	1.16 (0.46, 2.93)	4	0.74 (0.12, 4.52)
X-ray erosions at BL				
No (estimated 50%)	69	Reference	5	Reference
Yes (estimated 50%)	68	0.93 (0.42, 2.06)	4	0.69 (0.13, 3.54)
Latent class				
1 (estimated 39%)	65	Reference	5	Reference
2 (estimated 38%)	72	1.31 (0.57, 3.01)	3	0.81 (0.12, 5.51)
3 (estimated 23%)	69	1.28 (0.48, 3.45)	5	1.39 (0.19, 9.95)
Response at 6 months (*n* = 152)				
MUS synovitis at BL				
No (estimated 23%)	66	Reference	6	Reference
Yes (estimated 77%)	65	1.08 (0.40, 2.93)	9	1.79 (0.32, 9.97)
X-ray erosions at BL				
No (estimated 49%)	64	Reference	7	Reference
Yes (estimated 51%)	66	1.30 (0.55, 3.07)	10	1.78 (0.47, 6.79)
Latent class				
1 (estimated 40%)	62	Reference	8	Reference
2 (estimated 38%)	72	1.38 (0.56, 3.41)	3	0.50 (0.08, 3.04)
3 (estimated 22%)	59	1.21 (0.41, 3.56)	18	2.84 (0.64, 12.50)

Proportions of responders at 3 and 6 months shown by clinically significant ultrasound determined synovitis, baseline erosion and by combined clinical and ultrasound latent class at baseline. AE: adverse event, BL: baseline, MUS: musculoskeletal ultrasound; RRR: relative risk ratio.

**Figure 2.**
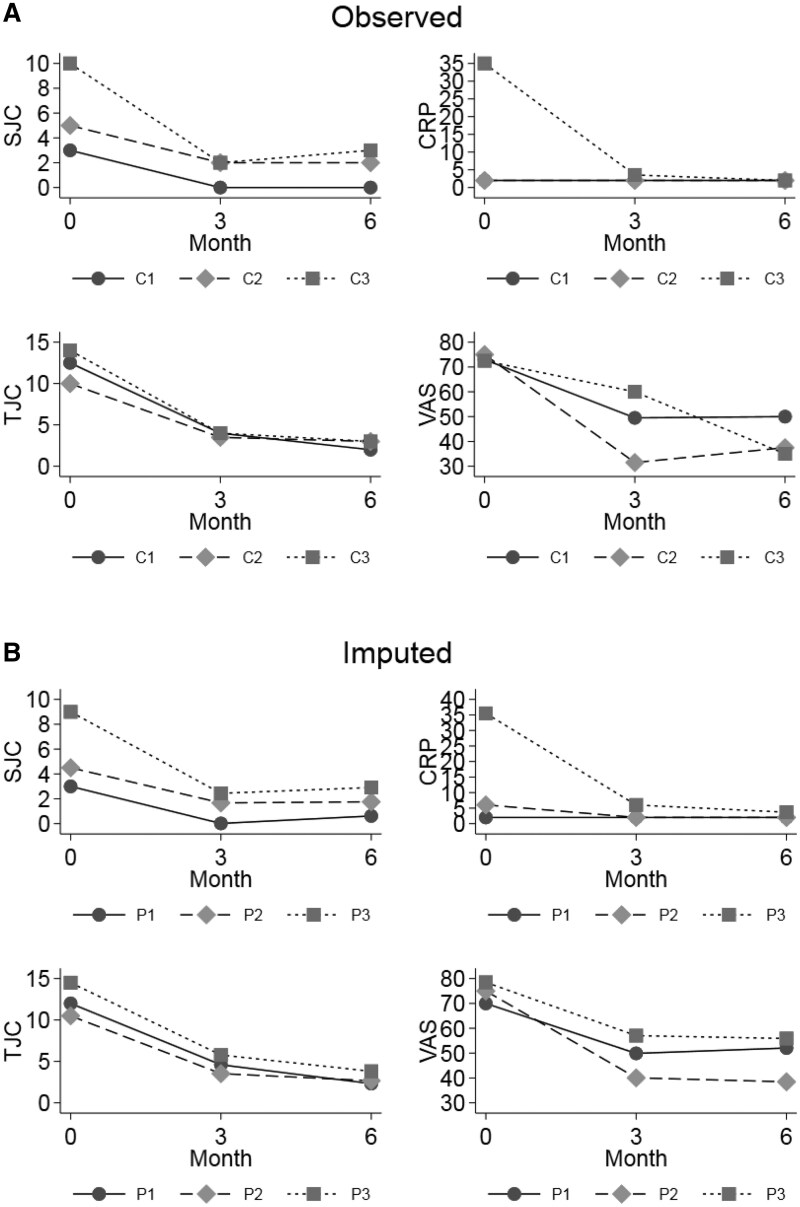
Median DAS28-CRP components over time for each cluster. Graphs showing median DAS28 components over 6 months for each cluster. (**A**) Observed data; (**B**) imputed data. C1: cluster 1; C2: cluster 2; C3: cluster 3; P1: cluster 1; P2: cluster 2; P3: cluster 3. SJC: swollen joint count; TJC: tender joint count; VAS: visual analogue score

**Figure 3.**
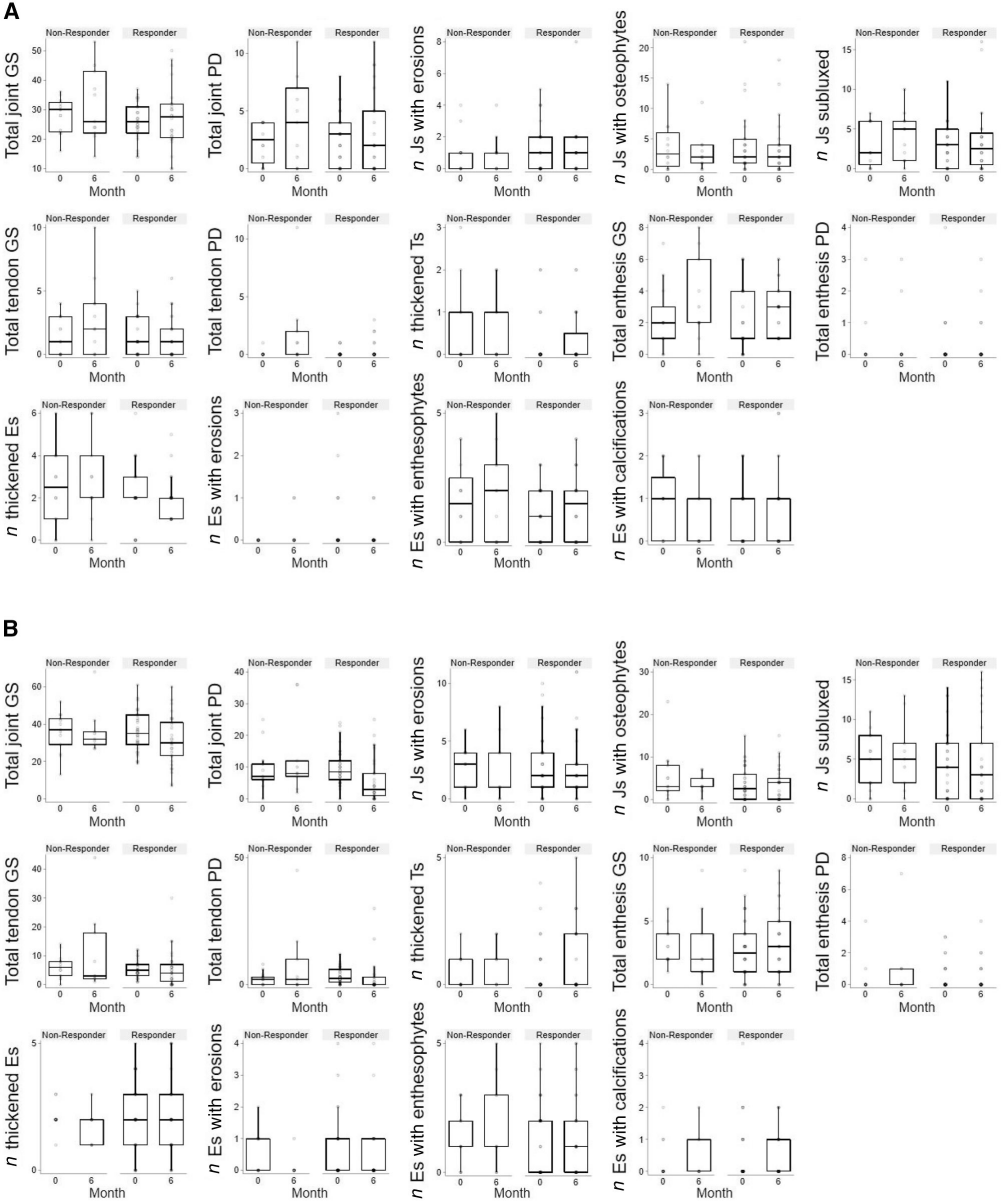

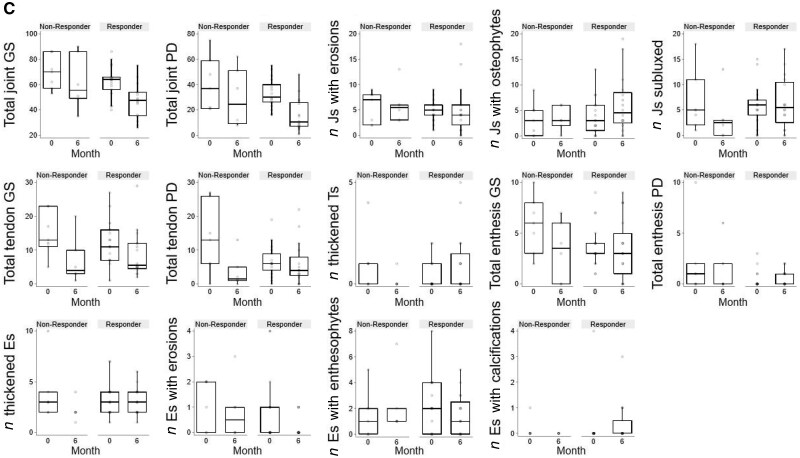
Ultrasound scores at baseline and 6 months. Bar chart showing ultrasound scores by response status at 6 months for each of the combined clinical and ultrasound classes 1–3. (**A**) Class 1. Non-responder: *n* = 11; responder: *n* = 24. (**B**) Class 2. Non-responder: *n* = 9; responder: *n* = 29. (**C**) Class 3. Non-responder: *n* = 6; responder: *n* = 16. E: enthesis; J: joint; GS: grey scale; PD: power Doppler; T: tendon

Fig. 2 shows the changes in the individual DAS28 components in clusters 1–3 for both the observed and the imputed data at 3 and 6 months. CRP and SJC improve more in cluster 3 (SJC to month 3), both variables higher at baseline. TJC improved in all the clusters similarly. Fig. 3 shows individual MSUS variables at baseline and 6 months by response status for each cluster. In cluster 1, with lowest baseline median MSUS values, most variables at baseline and 6 months for cluster 1 were not different between responders and non-responders. For cluster 2, a small improvement in joint and tendon PD in responders relative to non-responders was seen, but for most variables there was minimal difference between responders and non-responders. Cluster 3, with highest baseline joint GS and PD, showed greater improvement in these in responders than in non-responders. Improvement in tendon inflammation (GS and PD) was observed in both groups although appeared more marked in non-responders than responders.

## Discussion

To our knowledge, this study is the first to systematically evaluate whether presence of MSUS determined synovitis/tenosynovitis and/or clinical-MSUS defined phenotypes in a b/tsDMARD treated, established RA cohort associates with response to TT. Unlike most other MSUS studies, comprehensive (38 joint, 20 tendons/tendon groups and 10 entheseal site) ultrasound was performed alongside disease activity measures to identify clinical phenotypes. Three phenotypes were identified and MSUS confirmed low to high inflammatory clusters. Interestingly, neither presence of clinically significant ultrasound-detected baseline synovitis or baseline cluster was associated with clinical response to TT. HAQ-DI correlated with levels of inflammation, but anxiety, depression and fatigue were not significantly greater in the low inflammatory group.

Compared with previous studies that have identified phenotypes of RA [3, 4, 18–20], this study utilized core clinical measures that are used to assess response and inform treatment decisions in routine practice, and identify clinically meaningful clusters that may have direct application to clinical practice. Specifically, this study formally assessed whether the presence of ultrasound-confirmed synovitis before starting a new TT predicts subsequent response to treatment. Unexpectedly, no correlation was identified between the presence or absence of synovitis and response and the three distinct clinical-MSUS clusters identified in the present study also did not appear to associate with clearly different treatment responses.

Cluster 1 comprised a greater proportion of females, seronegative status and lower DAS28 subcomponents of inflammation (acute phase and SJC) than the other clusters, consistent with other studies [1, 2]. Good concordance was observed between the clinical subcomponents of the clusters and MSUS assessment of joint level inflammation. Every patient within high inflammatory cluster 3 had clinically relevant ultrasound-detected synovitis, whereas half of cluster 1 patients did not and greater levels of tenosynovitis from cluster 1 to cluster 3 were also observed. Cluster 3, with a more inflammatory profile, the longest disease duration and MSUS determined synovitis had the most joint erosions. Cluster 3 had evidence of greater active inflammation (PD) at the enthesis, typically recognized across spondyloarthritis (SpA) spectrum disease [21], whilst clusters 1 and 2 had higher number of enthesis calcifications. Seronegative RA that may overlap with SpA disease spectrum [22] may account for these observations, although the overall number with entheses was low in the entire cohort.

Several studies have shown low inflammatory phenotypes characterized by high PRO [3, 4] or higher levels of psychological comorbidity or fibromyalgia [18–20], which is also termed secondary (nociplastic) pain [23]. In this study, only functional limitation (HAQ-DI) was significantly increased in the more inflammatory cluster 3. There also appeared to be no meaningful difference in DAS-P, although analysis showed a significance for association with the lower inflammatory cluster. These findings appear contradictory to previous studies mentioned earlier [3, 4, 18–20]. Different populations, variables, PROs employed and clustering methods may account for this partly. Heterogeneity within low inflammatory cluster patients may exist and understanding pain across RA better and/or ongoing nociceptive input [23] may help make sense of these observations.

Clear improvement in GS and PD synovitis was observed, as expected, in patients who responded to therapy, with the most marked improvement in the more inflammatory cluster 3. With low baseline levels of inflammation in cluster 1, there was little margin for improvement in PD to differentiate ultrasound changes of responders and non-responders. Despite this, 69% of patients within cluster 1 met EULAR criteria for a moderate or good response to therapy. In cluster 3, non-responders also showed some improvement on MSUS, particularly in scores for tenosynovitis.

Thus, in all clusters, most patients responded to TT regardless of the relative amount of MSUS inflammation associated with that cluster. This challenges the conventional wisdom that all individuals with low inflammatory phenotypes, driven by more subjective DAS28 components do not comprise reversible drivers of disease activity and are unlikely to respond to therapy. MSUS also showed that the low inflammatory cluster 1 did not appear to be driven by damage, with no differences in joint damage, subluxation or osteophytosis across clusters.

A couple of reasons might explain these study findings. It is conceivable that TT has effects beyond improvement in joint synovitis and systemic inflammation. For example, IL-6 blockade has been shown to improve anaemia of chronic disease, improve sleep and has been associated with both weight gain and an improvement in muscle mass [24], all of which will serve to improve general health, with similar effects likely for other TTs. The use of JAKi appears to have secondary benefits also, with improvement in pain that is over and above their effects on inflammation and confers higher response rates when compared directly to bDMARDs [25, 26]. Such influences may account for an improvement in DAS28 in a subset of patients who have an absence of MSUS defined synovitis and/or an improvement in MSUS synovitis.

This study was pragmatic in design to represent standard UK clinical practice and lacked a non-treatment or placebo arm. However, it would be unethical to withhold treatment in an active, established RA cohort and the proportion of patients responding to treatment is persuasive, which at 60–70% is consistent with therapeutic response seen in many TT trials. The reduced follow-up cohort was a limitation and the analysis was based on a relatively modest cohort of patients and therefore may have lacked the power to identify all clusters of a much larger analysis such as using registry studies [3]. However, this study benefitted from comprehensive and prospective phenotyping of patients including extensive MSUS, providing a sense check of joint level disease activity.

Taken together, these data highlight the hazards of assessing disease activity and therapeutic response solely on measures considered to be more objective of RA disease pathology (such as SJC, CRP and the 2C-DAS28 and MSUS measures) and overlooking broader indicators of disease activity, including PRO. These findings also emphasize that our understanding of persistent PRO symptoms of pain and fatigue that typically drive PROs remains poor and that several subphenotypes are likely to exist.

In summary, three distinct patient phenotypes were identified, non-inflammatory to moderate inflammatory to high inflammatory, based on DAS28 components and MSUS synovitis. Importantly, this study confirmed that the presence of synovitis on MSUS and/or higher inflammatory cluster was not associated with a higher likelihood of treatment response and that change in MSUS measures of inflammation did not correlate fully with clinical response. In all clusters, TT improved PD synovitis but also appeared to improve measured clinical disease state even in those with minimal synovitis on MSUS, indicating TT efficacy working beyond simply resolving joint inflammation in RA patients. This warrants further investigation and has important implications for trial design going forwards, including eligibility and outcome measures of response.

## Supplementary Material

keaf315_Supplementary_Data

## Data Availability

All data relevant to the study are included in the article or uploaded as online supplementary information. Additional data are available on reasonable request.
