# Supplementary material for: Neither ultrasound synovitis nor clinical-ultrasound phenotypes of established rheumatoid arthritis predict response to targeted therapy
Source: Rheumatology (Oxford). 2025 Jun 25;64(12):6079–89. doi: 10.1093/rheumatology/keaf315 (PMC12671869; doi:10.1093/rheumatology/keaf315)
Supplement: keaf315_Supplementary_Data [file keaf315_supplementary_data.docx]

**Title:** Neither baseline ultrasound synovitis nor combined clinical-ultrasound defined phenotypes predict treatment response in an advanced therapy treated rheumatoid arthritis cohort

**Supplementary file**

**Statistical analysis**

Latent profile analysis assumes that there is an unmeasured (latent) categorical variable, i.e. two or more distinct phenotypical clusters and these give rise to differences in observed variables. When these observed variables are continuous rather than categorical, this analysis is deemed latent profile analysis rather than the more familiar latent class analysis.

Poisson and negative binominal models were also run and w ith this approach, for TJC, VAS and joint GS a normal distribution was specified, for enthesis hypo-echogenicity and thickening a Poisson distribution was chosen and for all other variables the negative binomial distribution worked best.

**Sample size**

Variables imputed were smoker status, presence of clinically relevant synovitis at baseline, baseline erosions, disease duration, baseline values for HADS anxiety and depression, FACIT-fatigue score and ESR, and TJC, SJC, CRP, and patient VAS at 3 and 6 months. The imputation models also included age, sex, RF, CCP, ANA,, the number of previous treatments, latent class membership at baseline (clinical and MSUS combined), and baseline values of TJC, SJC, CRP & patient VAS.

**Results**

| Enthesis | Measurement |
| --- | --- |
| lateral epicondyle enthesis/tendon | 6.4mm |
| patella ligament proximal insertion | 6.1mm |
| Patella ligament distal insertion | 4 mm |
| Achilles’ tendon | 5.29 mm |
| Plantar facia | 4.4 mm |

**Supplementary Table S1**: Standardised measurements for tendon thickening at entheses

| Number of previous TT | Number of patients (%) Total n=200 |
| --- | --- |
| 0 | 57 (28.5) |
| 1 | 46 (23) |
| 2 | 37 (18.5) |
| 3 | 26 (13) |
| 4 | 16 (8) |
| 5 | 11 (5.5) |
| 6 | 5 (2.5) |
| 7 | 2 (1) |

**Supplementary Table S2**: Number of patients and percentage of cohort exposed to each line of targeted therapy (TT)

| Number of previous classes | Number of patients (%)  Total n=200 |
| --- | --- |
| 0 | 57 (28.5) |
| 1 | 55 (27.5) |
| 2 | 40 (20.1) |
| 3 | 33 (16.58) |
| 4 | 12 (6.03) |
| 5 | 3 (1.51) |

**Supplementary Table S3**: Number of patients and percentage of cohort exposed to numbers of different classes of targeted therapy (TT)


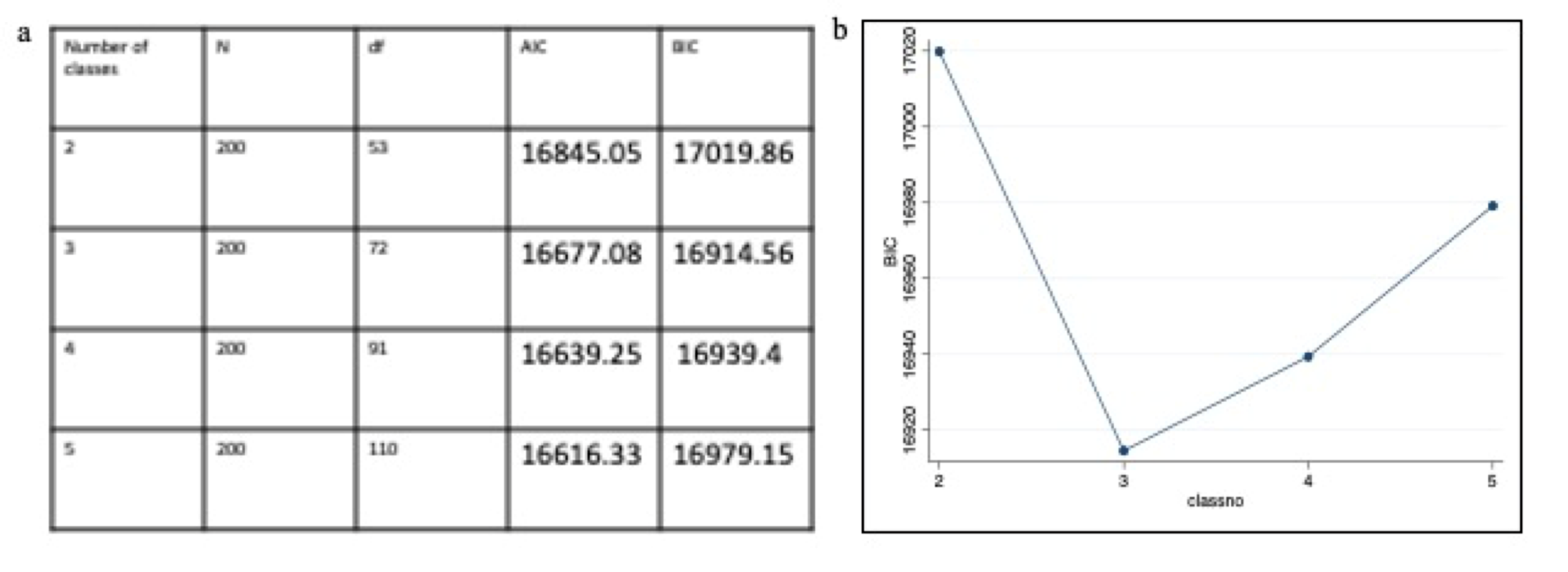


**Supplementary Figure S1**: Fit statistics for combined clinical and ultrasound variable latent profile model. a. table of results for AIC/BIC. b. Graph of BIC results for each number of classes.

*Df – degrees of freedom, AIC – Akaike Information Criterion, BIC – Bayesian Information Criterion.*

| Biologic prescribed | Cluster 1 | Cluster 2 | Cluster 3 | total |
| --- | --- | --- | --- | --- |
| Adalimumab | 8/76  (10.5%) | 24/81  (29.6%) | 8/42  (19.0%) | 40/199  (20.1%) |
| Etanercept | 6/76  (7.9%) | 4/81  (4.9%) | 3/42  (7.1%) | 13/199  (6.5%) |
| Tocilizumab | 5/76  (6.6%) | 8/81  (9.9%) | 6/42  (14.3%) | 19/199  (9.5%) |
| Rituximab | 7/76  (9.2%) | 13/81  (16.0%) | 4/42  (9.5%) | 24/199  (12.1%) |
| Abatacept | 11/76  (14.5%) | 9/81  (11.1%) | 1/42  (2.4%) | 21/199  (10.6%) |
| Baricitinib | 29/76  (38.2%) | 19/81  (23.5%) | 17/42  (40.5%) | 65/199  (32.7%) |
| Other | 10/76  (13.2%) | 4/81  (4.9%) | 3/42  (7.1%) | 17/199  (8.5%) |

**Supplementary Table S4:** *Targeted therapies prescribed for the study by cluster. Other – Infliximab, Golimumab, Certolizumab, Sarilumab.*

| Characteristic | Difference between latent profile (p value) | | |
| --- | --- | --- | --- |
|  | 1/2 | 1/3 | 2/3 |
| Age | 0.2240 | **0.0016** | **0.0095** |
| Disease duration | 0.1915 | **0.0353** | **0.0047** |
| TJC | 0.2569 | **0.0092** | **0.0017** |
| SJC | **0.0000** | **0.0000** | **0.0000** |
| CRP | **0.0000** | **0.0000** | **0.0000** |
| ESR | **0.0025** | **0.0000** | **0.0000** |
| HAQ | 0.3894 | **0.0077** | **0.0037** |
| DAS-P | **0.0014** | **0.0000** | **0.0010** |
| DAS28CRP-2C | **0.0000** | **0.0000** | **0.0000** |

**Supplementary Table S5**: Dunn test results for clinical variables with statistically significant differences between clusters in the combined clinical and ultrasound model. Significant results highlighted in bold.

| Characteristic | Differences between latent profiles (p value) | | |
| --- | --- | --- | --- |
|  | 1/2 | 1/3 | 2/3 |
| Total joint B mode | **0.0000** | **0.0000** | **0.0000** |
| Total joint Doppler | **0.0000** | **0.0000** | **0.0000** |
| Total joint erosions | **0.0001** | **0.0000** | **0.0000** |
| Total tendon B mode | **0.0000** | **0.0000** | **0.0000** |
| Total tendon Doppler mode | **0.0000** | **0.0000** | **0.0000** |
| Total tendon thickening | **0.0199** | **0.0168** | 0.3364 |
| Total Enthesis hypogenicity | 0.2336 | **0.0008** | **0.0047** |
| Total enthesis Doppler | 0.1205 | **0.0011** | **0.0164** |
| Total enthesis thickening | 0.1378 | **0.0010** | **0.0128** |
| Total enthesis erosions | **0.0237** | **0.0000** | **0.0037** |
| Total enthesis calcification | 0.0879 | **0.0002** | **0.0060** |

**Supplementary Table S6**: Dunn test results for ultrasound variables with statistically significant differences between clusters. Significant results highlighted in bold.
